# 

**Published:** 1901-04

**Authors:** 


### PENNSYLVANIA PERSONALS.

The many friends of Drs. Thomas B. and James B. Rayner will be glad to learn of their improved health and ability to be in attendance at the State meeting of Pennsylvania.

Dr. Jacob Helmer, of Scranton, is one of the most advanced advocates of better municipal milk-inspection in the country.

Pennsylvania's association knows no rival in the thorough discussion of papers and subjects on the convention floors. Her members are taught to discuss, interrogate, and relate their experiences, hence the remarkable attendance every year.

Ex-President Hurley, of the Veterinary Medical Association, of New Jersey, was among the delegates from that State to the convention.

Every one was glad to see Hoskins' double, Dr. Phillips, of North East, Pa., the great grape-raiser of the profession.

Dr. Ranck, as corresponding secretary, should receive a hearty vote of thanks of the profession for so excellent a programme he so much aided in securing for the annual meeting.

Dr. James M. Mecray was another of the New Jersey veterinarians in attendance at the meeting.

Dr. J. C. McNeil was there from the "Smoky City" to advocate its shady beauties and midnight attractions for the members in the semi-annual meeting of 1901.

Dr. James L. Robertson, of New York City, was prevented from being in attendance by an attack of "grippe," confining him to his home for two weeks.

President Harger should be a very happy man indeed for the extremely successful meeting of his first administration.

The morning clinics at the Veterinary Department of the University of Pennsylvania were taken advantage of by visiting members during the week.

Dr. D. K. Light, of Lebanon, of the class of 1880, at the American Veterinary College, was among the visitors from Southern Pennsylvania.

Dr. H. W. Read, of Freehold, N. J., was a delegate from New Jersey, and in attendance at both days of the convention. Dr. C. H. Magill was also present from New Jersey.

